# Supplementary figures and images for: Aberrant induction of p19Arf-mediated cellular senescence contributes to neurodevelopmental defects
Source: PLoS Biol. 2022 Jun 14;20(6):e3001664. doi: 10.1371/journal.pbio.3001664 (PMC9197032; doi:10.1371/journal.pbio.3001664)

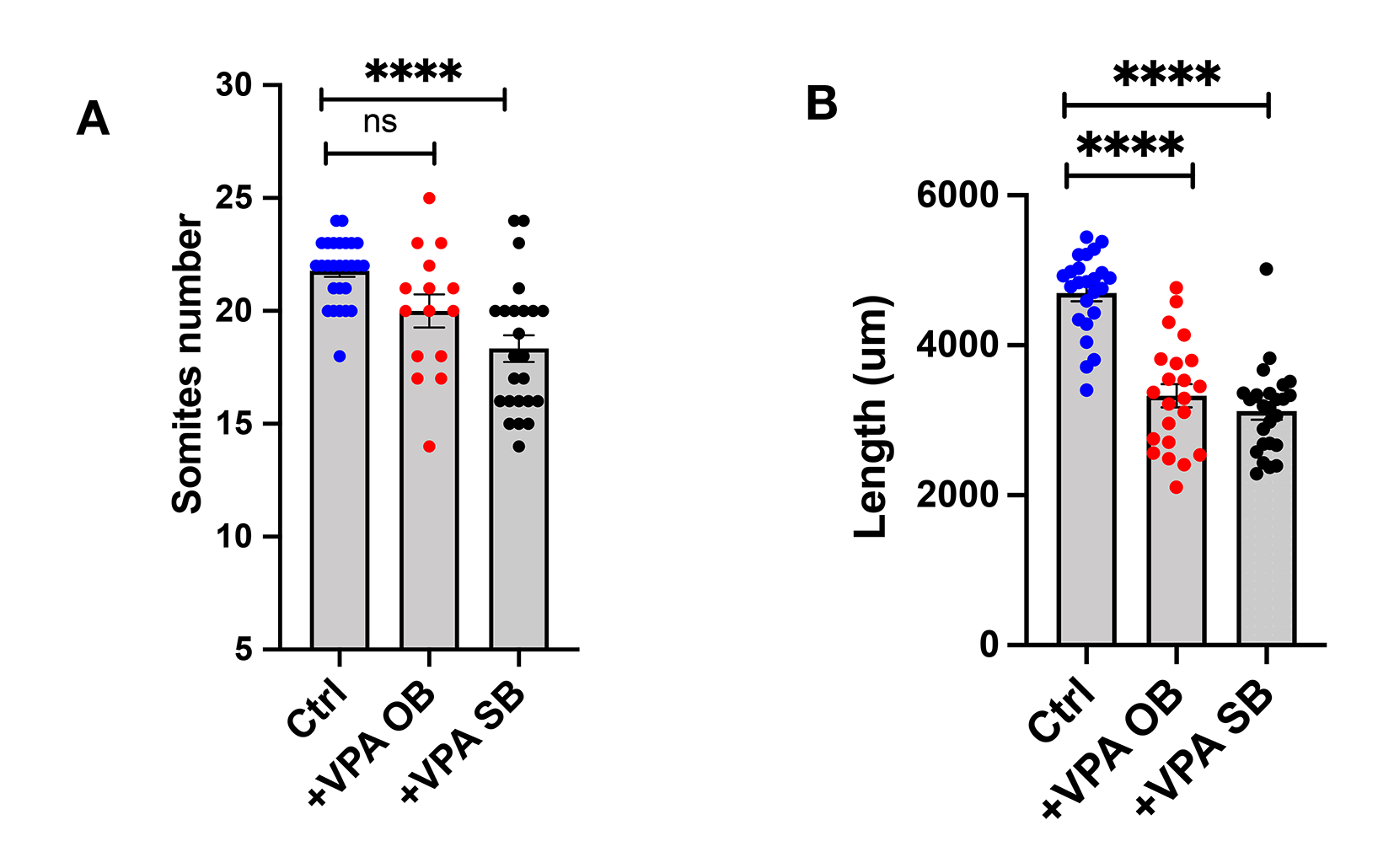

Supplement: S1 Fig — (A) Quantification of visibly intact somite number (Control, n = 27 from 12 litters; (OB), Open brain, n = 22 from 10 litters; (SB) Small brain, n = 25 from 16 litters). Data bars represent mean ± SEM. Kruskal–Wallis test: ns, no significant and ***p ≤ 0.001. (B) Measurements of the length of the embryo (from the otic vesicle to the tail tip) (Control, n = 24 from 12 litters; Open brain, n = 22 from 10 litters; Small brain, n = 25 from 16 litters. Data bars represent mean ± SEM. Kruskal–Wallis test: ns, no significant and ****p ≤ 0.0001. The data underlying this figure can be found in S1 Data. VPA, valproic acid. (TIF) [file pbio.3001664.s001.tif]

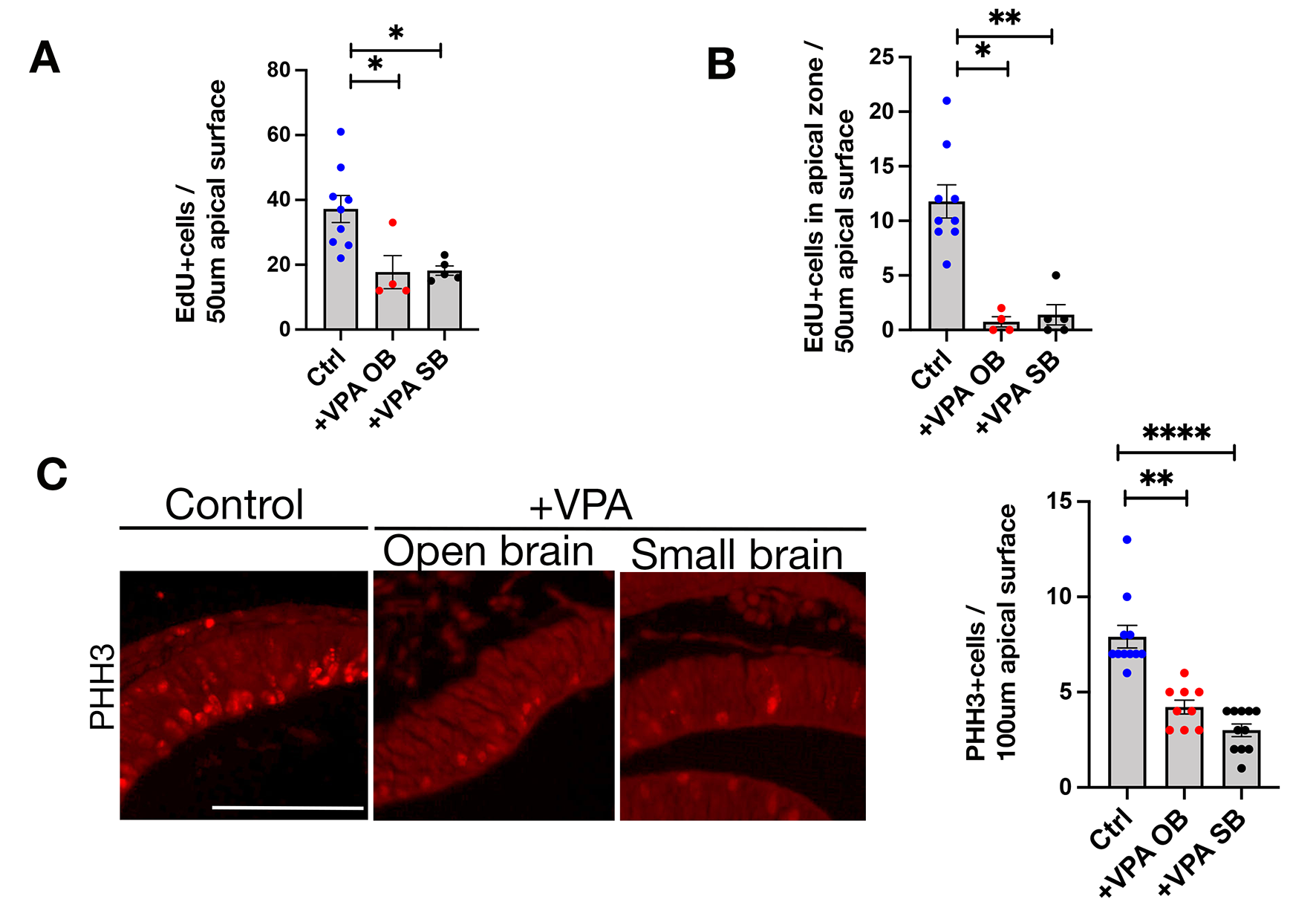

Supplement: S2 Fig — (A) Quantification of total EdU positive cells present at E9.5 (Control, n = 9 from 5 litters; Open brain, n = 4 from 3 litters; Small brain, n = 5 from 3 litters). Data bars represent mean ± SEM. Kruskal–Wallis test: *p ≤ 0.05. (B) Quantification of EdU positive cells in the apical zone at E9.5 (Control, n = 9 from 5 litters; Open brain, n = 4 from 3 litters; Small brain, n = 5 from 3 litters). Data bars represent mean ± SEM. Kruskal–Wallis test: *p ≤ 0.05 and **p ≤ 0.01. (C) Left: Immunostaining on sections of control and VPA-treated embryos for PHH3 (red) at E9.5. The square indicates the counted area. Scale bar, 100 μm. Right: PHH3 positive cells quantification at E9.5. (Control, n = 4 from 2 litters; Open brain, n = 3 from 3 litters; Small brain, n = 4 from 3 litters from 3 litters); 3 levels have been counted per embryo. Data bars represent mean ± SEM. Kruskal–Wallis test: **p ≤ 0.01 and ****p ≤ 0.0001. The data underlying this figure can be found in S1 Data. E, embryonic day; PHH3, phospho-histone H3; VPA, valproic acid. (TIF) [file pbio.3001664.s002.tif]

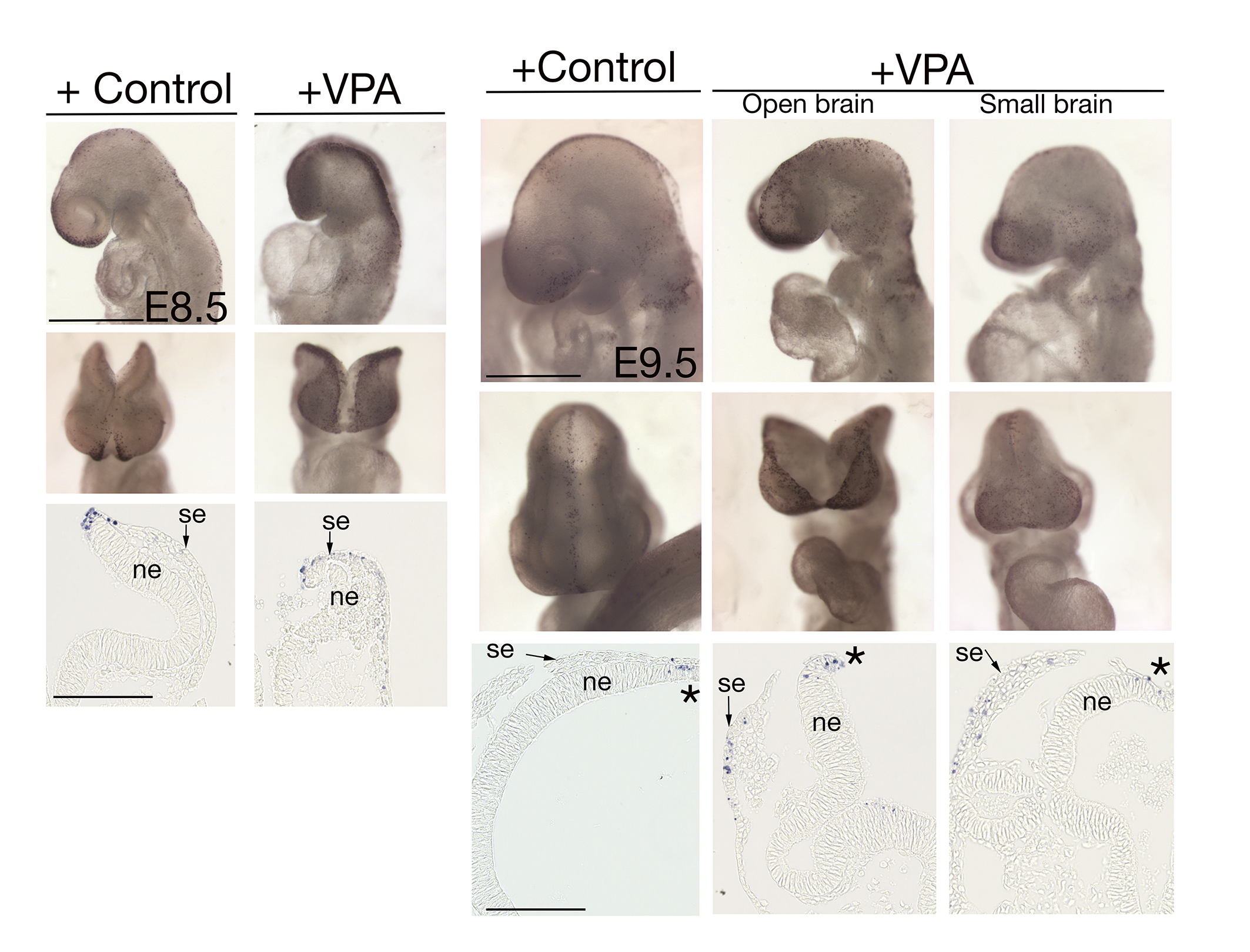

Supplement: S3 Fig — Control and VPA-treated embryos were stained with whole mount TUNEL assay, to assess cell death. (Left) Lateral views and frontal views of control and VPA-treated embryos dissected at E8.5. Scale bar, 500 μm. Corresponding horizontal sections at the forebrain level (3 embryos from at least 2 litters were analyzed). Scale bar, 100 μm. (Right) Lateral views and frontal views of control and VPA-treated embryos dissected at E9.5 (6 embryos from at least 5 litters were analyzed). Scale bar, 500 μm. Corresponding horizontal sections at the forebrain level. Scale bar, 100 μm. Some apoptotic cells are observed in the surface ectoderm. Positive cells are seen in the neural fold tips in all conditions (asterisk). E, embryonic day; ne, neuroepithelium; se, surface ectoderm; VPA, valproic acid. (TIF) [file pbio.3001664.s003.tif]

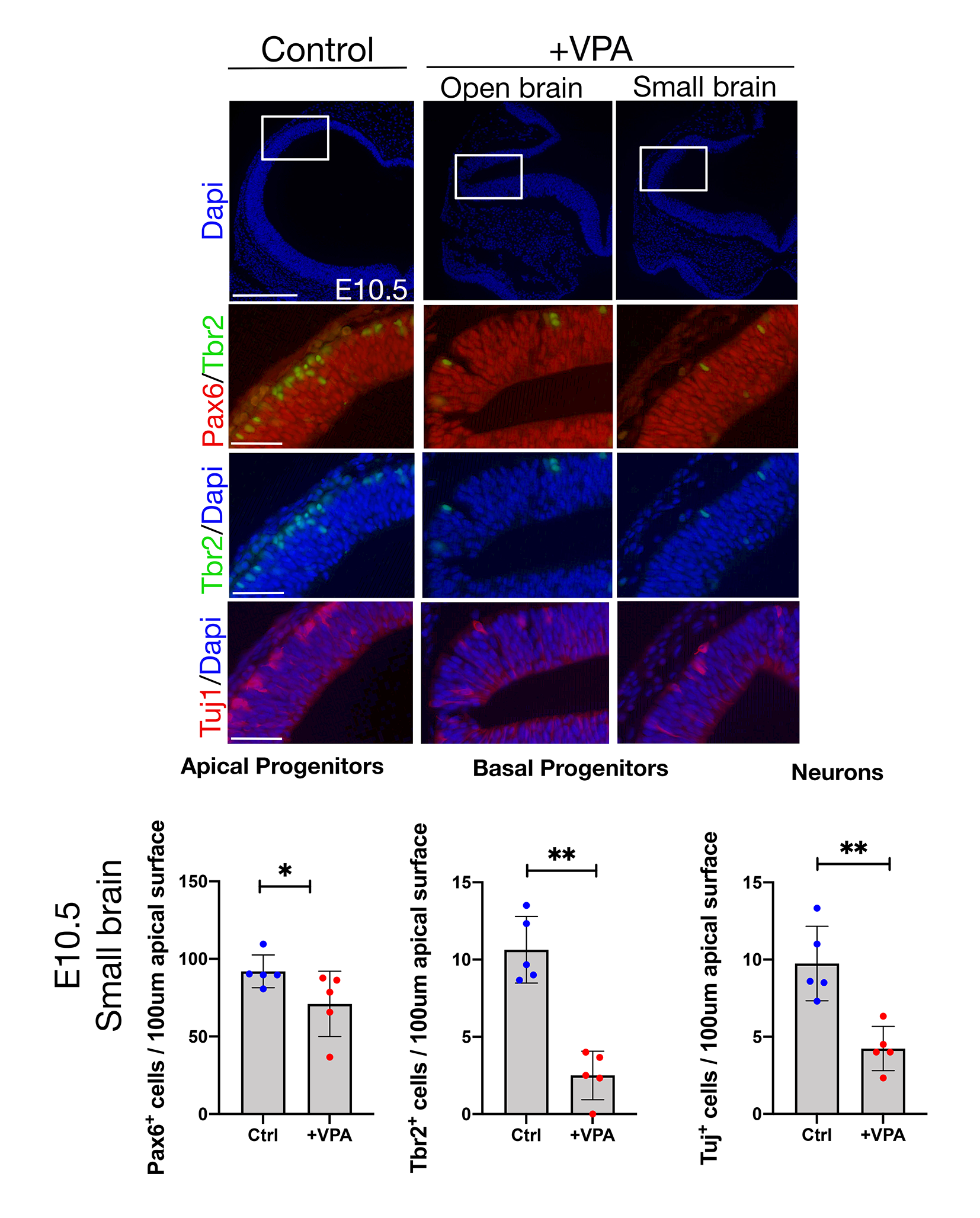

Supplement: S4 Fig — Cortical sections (coronal) of E10.5 embryos were immunoassayed for Pax6, Tbr2, Tuj1, and counterstained with Dapi. Scale bar, 250 μm (top row), 50 μm. Graphs show quantification of Pax6 and Tbr2 positive progenitors or the thickness of the neuronal layer in the microcephalic cortical vesicles (5 embryos from at least 4 different mothers were analyzed). Data bars represent mean ± SEM Mann–Whitney test: *p ≤ 0.05 and **p ≤ 0.01. The data underlying this figure can be found in S1 Data. E, embryonic day; VPA, valproic acid. (TIF) [file pbio.3001664.s004.tif]

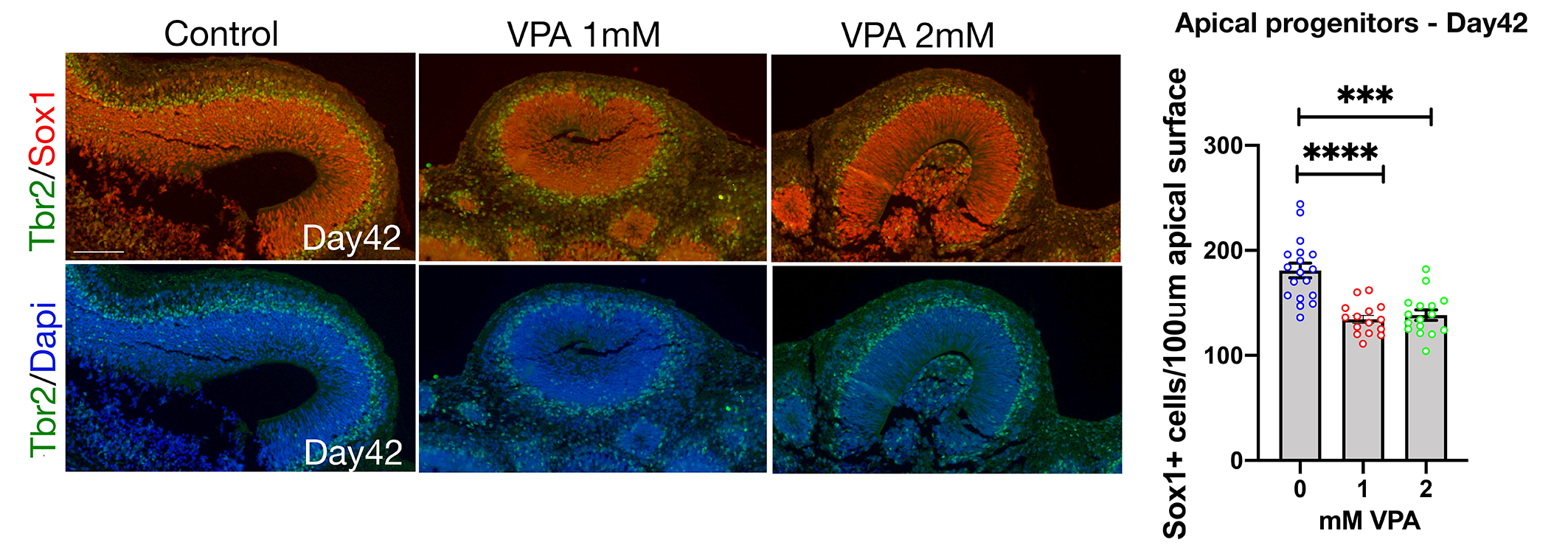

Supplement: S5 Fig — Sections through control and VPA-treated organoids were immunostained with Sox1(red), Tbr2 (green), and Dapi (blue) at day 42 (scale bar, 50 μm), (n = 15 (Control), 12 (1 mM VPA), 13 (2 mM VPA), 4 independent experiments). Kruskal–Wallis test: ***p ≤ 0.001 and ****p ≤ 0.0001. The data underlying this figure can be found in S1 Data. VPA, valproic acid. (TIF) [file pbio.3001664.s005.tif]

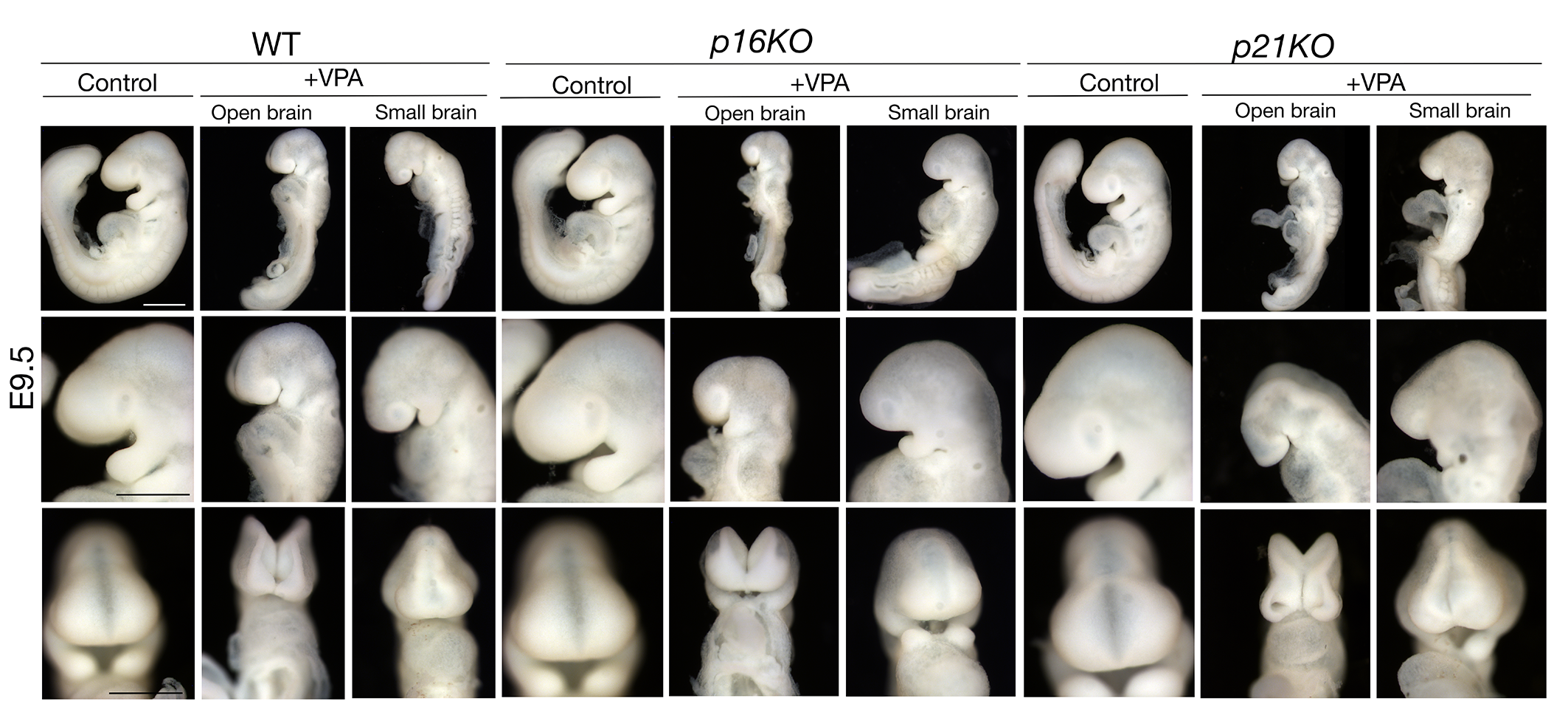

Supplement: S6 Fig — Lateral views of control and VPA-treated embryos deficient for p16Ink4a or p21 (top row). Scale bar, 500 μm. Higher magnification of the heads in lateral (middle row) and frontal views (bottom row). Scale bar, 500 μm. An open neural tube or a smaller brain, as well as a gross misalignment of the neural tube and somites are still observed after VPA treatment in the absence of p16Ink4a or p21. VPA, valproic acid. (TIF) [file pbio.3001664.s006.tif]

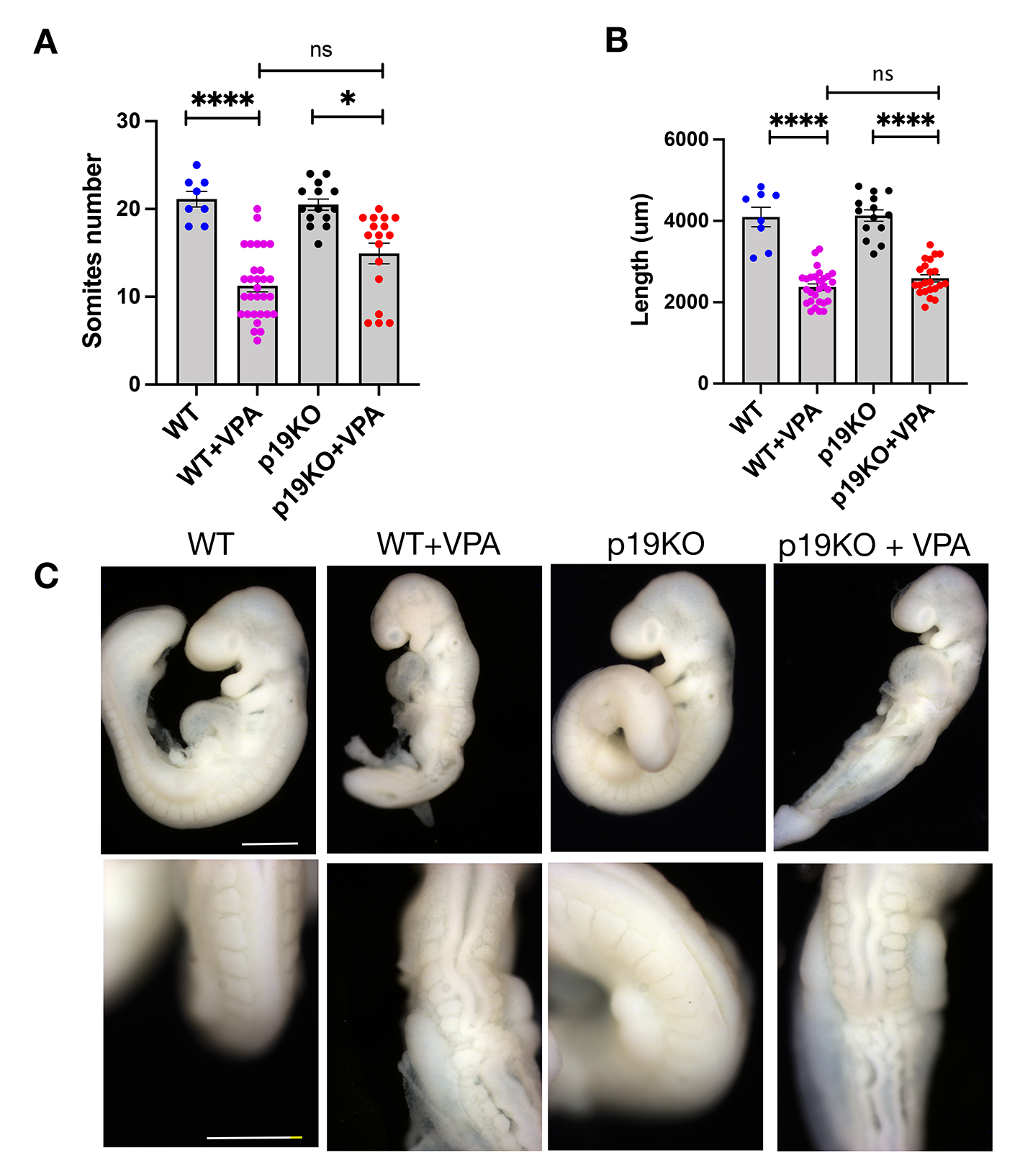

Supplement: S7 Fig — (A) Quantification of visibly intact somite number (WT, n = 8 embryos from 4 litters, WT+VPA, n = 30 embryos from 11 litters, p19KO, n = 14 embryos from 4 litters, p19KO + VPA, n = 17 embryos from 8 litters). Data bars represent mean ± SEM. Kruskal–Wallis test: ns, no significant, *p ≤ 0.05 and ****p ≤ 0.0001. (B) Measurements of the length of the embryo (from the otic vesicle to the tail tip (WT, n = 8 embryos from 4 litters, WT+VPA, n = 28 embryos from 11 litters, p19KO, n = 14 embryos from 4 litters, p19KO + VPA, n = 22 embryos from 8 litters). Data bars represent mean ± SEM. Kruskal–Wallis test: ns, not significant and ****p ≤ 0.000. (C) Lateral views (top) and dorsal views (bottom) of control and VPA-treated embryos dissected at E9.5, illustrating the pronounced curve in the neural tube and abnormally shaped somites observed (control embryo is same as shown in S6 Fig). Scale bar, 500 μm. The data underlying this figure can be found in S1 Data. E, embryonic day; VPA, valproic acid; WT, wild-type. (TIF) [file pbio.3001664.s007.tif]

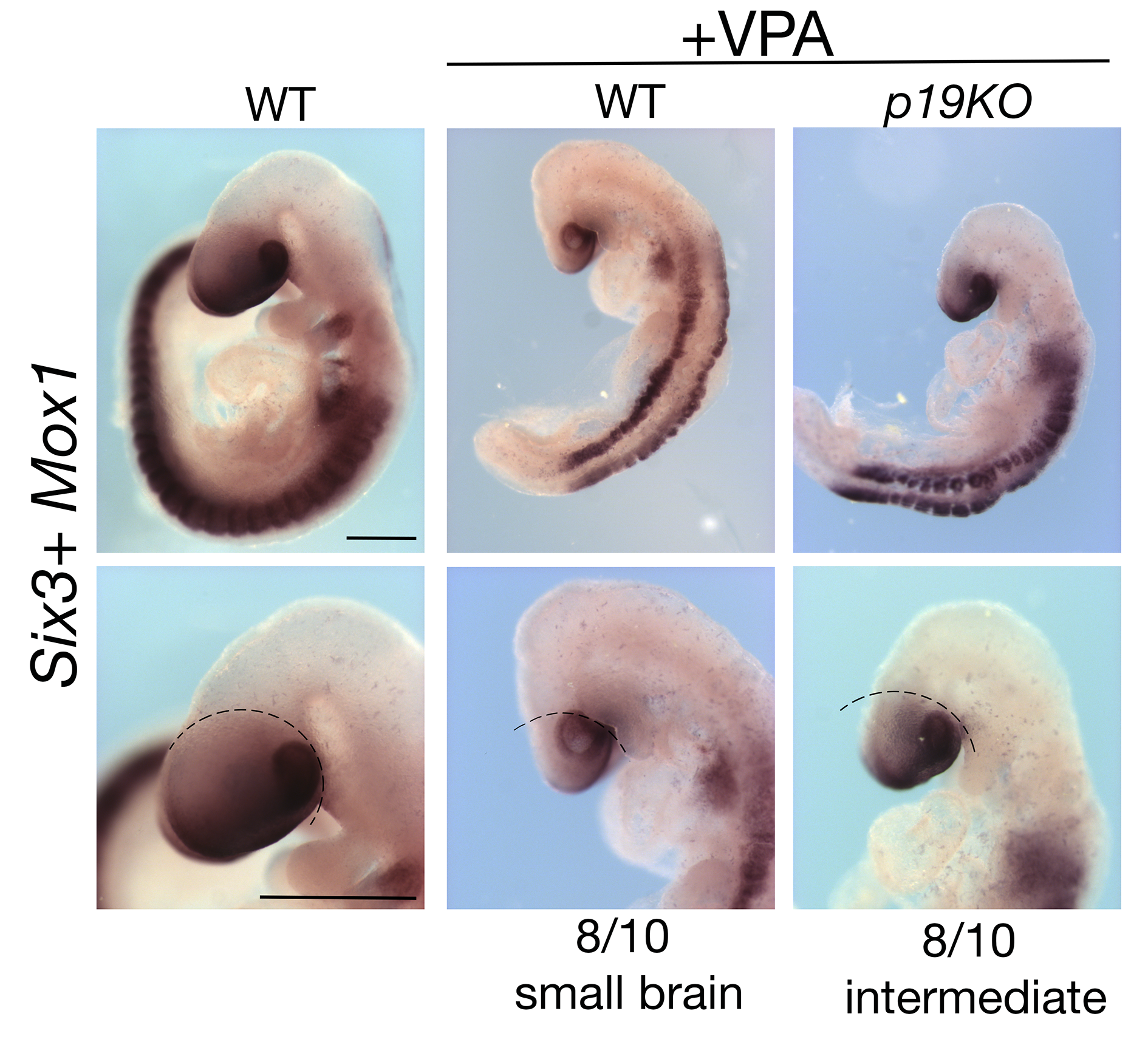

Supplement: S8 Fig — Whole mount in situ hybridization for Six3 (forebrain) and Mox1 (somites), showing an increased size of the forebrain in p19Arf-deficient, VPA-treated mice in comparison to the WT mice treated with VPA. Scale bar, 500 μm (top row) and 50 μm (bottom row). The number of embryos examined are indicated (n = 10 from at least 5 different litters). VPA, valproic acid; WT, wild-type. (TIF) [file pbio.3001664.s008.tif]

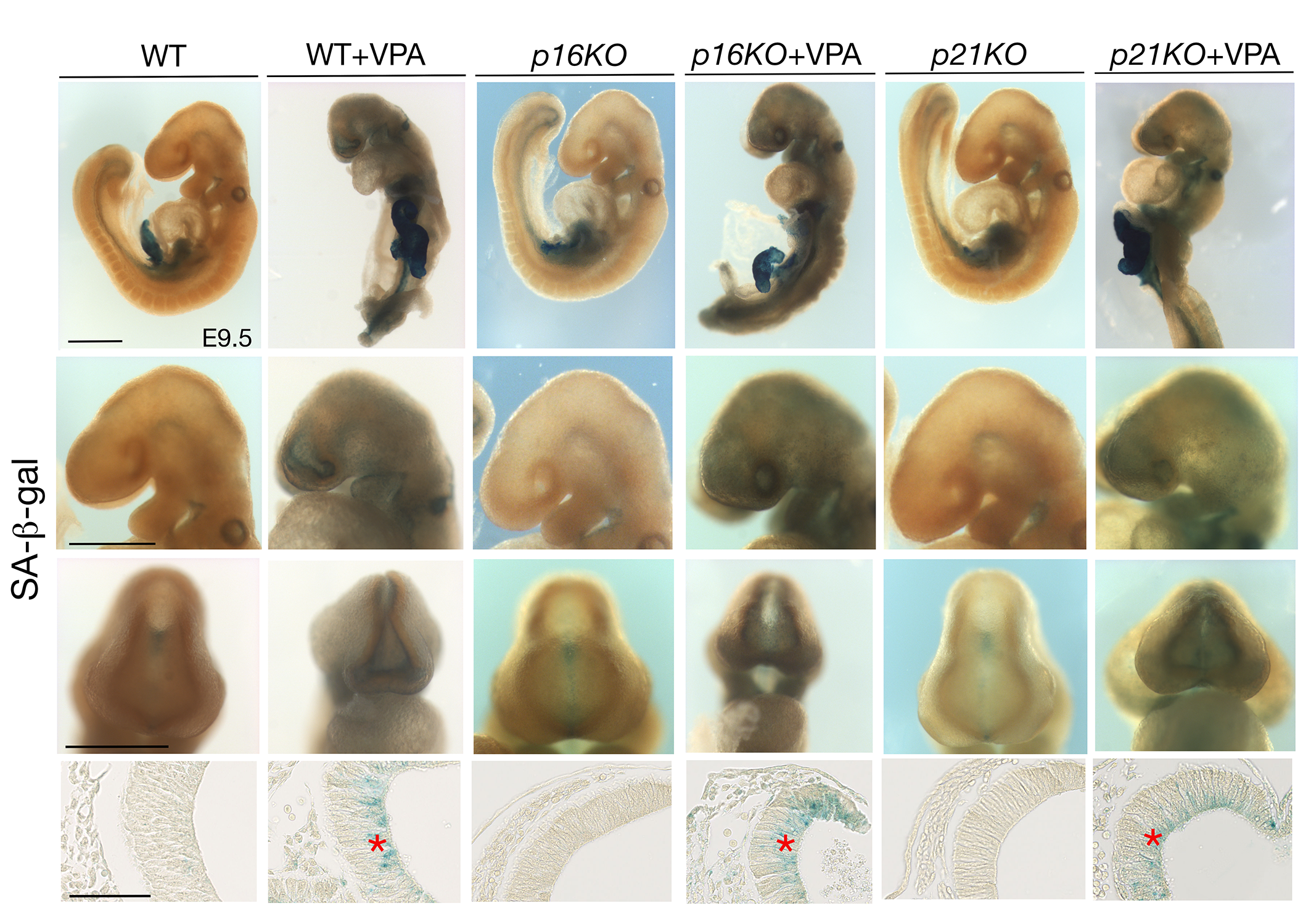

Supplement: S9 Fig — Whole mount SA-β-gal staining in control and VPA-treated embryos with small-brain phenotypes at E9.5 (WT, n = 9 embryos from 4 litters, WT+VPA, n = 10 embryos from 5 litters, p16KO, n = 2 embryos from 1 litter, p16KO + VPA, n = 10 embryos from 5 litters, p21KO, n = 6 embryos from 3 litters, p21KO + VPA, n = 5 embryos from 2 litters). Scale bar, 500 μm. Higher magnification of the heads in lateral (second row) and frontal views (third row). Scale bar, 50 μm. Bottom row, Sections through whole mount SA-β-gal stained forebrains (scale bar, 100 μm). Red asterisks highlight senescent cells. (WT, n = 5 embryos from 3 litters, WT+VPA, n = 10 embryos from 5 litters, p16KO, n = 2 embryos from 1 litter, p16KO + VPA, n = 10 embryos from 5 litters, p21KO, n = 6 embryos from 3 litters, p21KO + VPA, n = 5 embryos from 2 litters). E, embryonic day; WT, wild-type. (TIF) [file pbio.3001664.s009.tif]

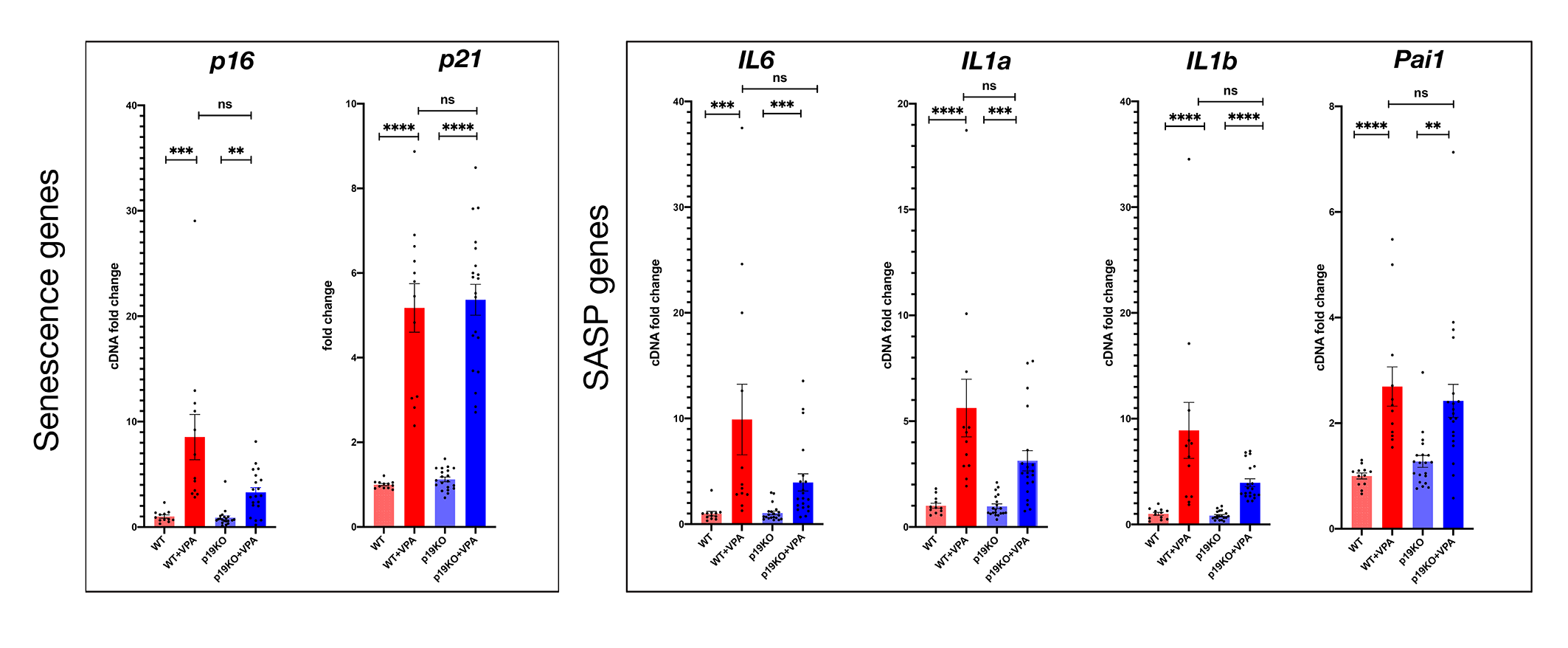

Supplement: S10 Fig — qRT-PCR analysis on E8.75 forebrain and midbrain, from control and p19Arf-deficient mice, treated with VPA or left untreated. Graphs show fold change expression for the senescence markers (p21 and p16Ink4a) and for SASP genes (IL6, IL1a, IL1b, and Pai1), normalized to untreated control (n = 12 (Control), n = 12 (Control+VPA), n = 20 (p19KO), n = 20 (p19KO+VPA), from at least 3 different litters). Data bars represent mean ± SEM. Kruskal–Wallis test: ns, no significant, **p ≤ 0.01, ***p ≤ 0.001 and ****p ≤ 0.0001. The data underlying this figure can be found in S1 Data. E, embryonic day; qRT-PCR, quantitative real-time PCR; SASP, senescence-associated secretory phenotype; VPA, valproic acid. (TIF) [file pbio.3001664.s010.tif]

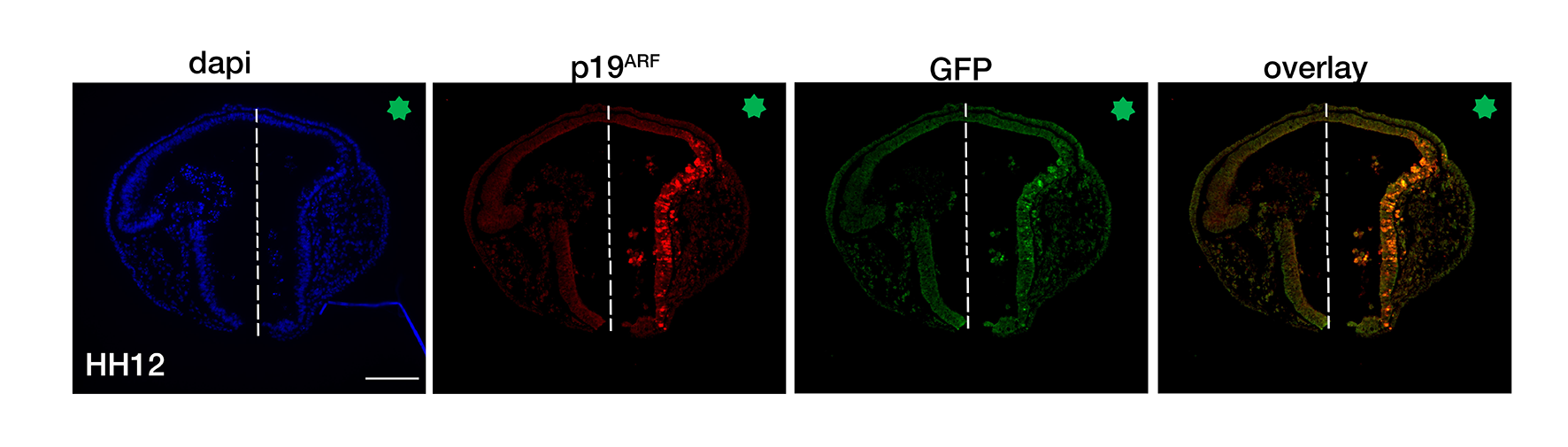

Supplement: S11 Fig — Sections through the neural tube of p19Arf-GFP electroporated chicken embryos electroporated at stage HH12, immunostained for p19Arf (red) and GFP (green), with Dapi counterstaining (blue). The green star shows the electroporated side. Scale bar, 100 μm. (TIF) [file pbio.3001664.s011.tif]

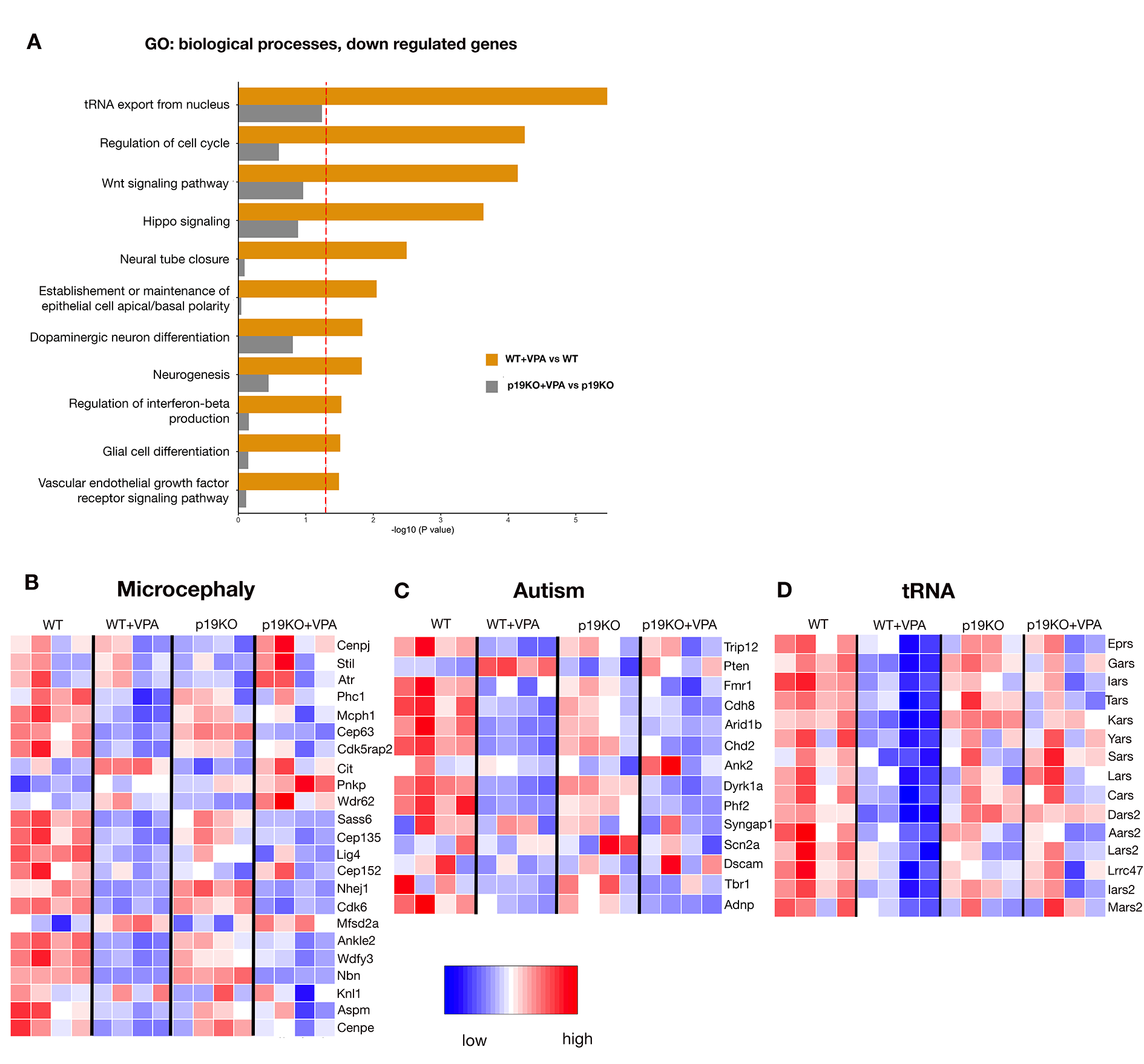

Supplement: S12 Fig — (A) GO Biological Processes pathway analysis on the down-regulated genes from RNA-seq of the forebrain and midbrain. Heat maps showing the relative expression of representative genes associated with (B) microcephaly (list generated from [35]), (C) autism (list generated from [35]) and (D) tRNA (list of genes identified in Fig 6C pathway analysis). The data underlying this figure can be found in S1 Data. RNA-seq, RNA sequencing; VPA, valproic acid. (TIF) [file pbio.3001664.s012.tif]
