# Supplementary material for: Aberrant induction of p19Arf-mediated cellular senescence contributes to neurodevelopmental defects
Source: PLoS Biol. 2022 Jun 14;20(6):e3001664. doi: 10.1371/journal.pbio.3001664 (PMC9197032; doi:10.1371/journal.pbio.3001664)
Supplement: S1 Table — (DOCX) [file pbio.3001664.s013.docx]

***Supplementary Table 1***

| **Human primers** | **Sequence** |
| --- | --- |
| GAPDH-fwd | AAGGTGAAGGTCGGAGTCAAC |
| GAPDH-rev | GGGGTCATTGATGGCAACAATA |
| p14^ARF -^fwd | CTGATGCTACTGAGGAGCCA |
| p14^ARF -^rev | TCATGACCTGGTCTTCTAGG |
| p16INK4A-fwd | GGTCGGAGGCCGATCCAGGTCA |
| p16INK4A-rev | TTCAATCGGGGATGTCTGAGG |
| p21-fwd | CGAAGTCAGTTCCTTGTGGAG |
| p21-rev | CATGGGTTCTGACGGACAT |
| IL1a-fwd | ACTGCCCAAGATGAAGACCAA |
| IL1a-rev | CCGTGAGTTTCCCAGAAGAAGA |
| IL1b-fwd | TTCGACACATGGGATAACGAGG |
| IL1b-rev | TTTTTGCTGTGAGTCCCGGAG |
| IL8-fwd | ACTGAGAGTGATTGAGAGTGGAC |
| IL8-rev | AACCCTCTGCACCCAGTTTTC |
| PAI1-fwd | AAGATCGAGGTGAACGAGAGTG |
| PAI1-rev | GACCACAAAGAGGAAGGGTCT |
|  |  |
| **Mouse primers** | **Sequence** |
| Rplp0 | QT00249375(QIAGEN) |
| P19^Arf^ | QT01164891 (QIAGEN) |
| p16^Ink4a^ | QT01164898(QIAGEN) |
| p21 | QT00137053(QIAGEN) |
| Il1a-fwd | GCACCTTACACCTACCAGAGT |
| Il1a-rev | AAACTTCTGCCTGACGAGCTT |
| Il1b-fwd | TTCAGGCAGGCAGTATCACTC |
| Il1b-rev | GAAGGTCCACGGGAAAGACAC |
| Il6-fwd | TAGTCCTTCCTACCCCAATTTCC |
| Il6-rev | TTGGTCCTTAGCCACTCCTTC |
| Pai1-fwd | TGGGTGGAAAGGCATACCAAA |
| Pai1-rev | AAGTAGAGGGCATTCACCAGC |
| MMP9-fwd | GCGTCATTCGCGTGGATAAG |
| MMP9-rev | TGGAAACTCACACGCCAGAA |

**Table S1. Primers used for qRT-PCR in the study.** The table lists the primer sequences or source for both human and mouse genes used in the study.
