# Supplementary material for: Aberrant induction of p19Arf-mediated cellular senescence contributes to neurodevelopmental defects
Source: PLoS Biol. 2022 Jun 14;20(6):e3001664. doi: 10.1371/journal.pbio.3001664 (PMC9197032; doi:10.1371/journal.pbio.3001664)
Supplement: Attachment — Submitted filename: Respone to reviewers.pdf [file pbio.3001664.s015.pdf]

Dear Lucas,

Thank you very much for handling the submission of our manuscript entitled "***Aberrant induction of p19^{Arf}-mediated cellular senescence contributes to neurodevelopmental defects***" (PBIOLOGY-D-21-02829R1). We were pleased with the very positive and useful comments from the reviewers, whose suggestions have definitely helped to improve the manuscript. We have attempted to address all of their suggestions, either with additional experiments, additional quantification, text clarification or additional explanation. As a result, we believe the manuscript is significantly stronger and clearer for these suggestions, and we hope you will find it suitable for publication.

If you have any additional questions, or would like to discuss any aspect, I remain available.
Sincerely,
Bill

REVIEWS:

Reviewer #1: Rhinn et al test whether VPA-induced developmental defects involves senescence. They characterise the embryonic phenotype in mice, showing the VPA treatment induces malformation such as exencephaly, microcephaly and spinal defects, as well as defective neurogenesis in those affected tissues. They further show that VPA induces ectopic senescence in neuroepithelial cells during developmental neurogenesis. Genetically, they show evidence that p19 is sufficient and partially necessary for microcephaly induction. The data quality seems high and senescence assays are thorough. This is an interesting and highly original study, reinforcing the critical role of senescence in embryonic development. I only have a few rather minor points as follows.

We thank the reviewer for their appreciation and interest in our study.

They find no improvement in the VPA-phenotype in p21- or p16-defective embryos. How about the senescence phenotype? Do these embryos still show VPA-associated senescence or do the developmental defects appear despite senescence reduction?

This is a very interesting and pertinent question. While we show that p19-deficient mice have less senescence induction and a rescue in the VPA-induced microcephaly phenotype, to support the causative link with senescence, it is important to show that the p21- and p16-deficient mice, while having no phenotype rescue, also have no change in senescence induction. To address this, we have performed additional experiments to treat p21- and p16-deficient litters with VPA, and assess SA-β-Gal staining, as performed in the manuscript for the p19-deficient embryos. We include these new results (Supp. Fig. 9) showing that indeed, both p21- and p16-deficient embryos have the same phenotype in response to VPA as the wildtype mice, and have a similar induction of senescence, supporting our initial results that this phenotype is primarily mediated by p19^{Arf}.

This might be beyond the scope and results may not be decisive, but I am curious if they see increased histone acetylation in VPA-treated embryos and/or organoids. Do they see a similar effect with other HDACi in organoids?

This is an interesting question, which we agree is slightly beyond the scope of this manuscript, but which we also feel has been significantly addressed in the literature.

Numerous studies demonstrate that VPA is a potent HDAC-inhibitor (HDACi), similar to TSA and other HDACi's. These studies show that both lead to significantly increased histone acetylation.

In addition, previous studies show how both TSA and VPA can increase expression of senescence markers, including p19^{Arf}. Therefore, for many aspects, both drugs function as HDACi's that can cause acetylation and senescence induction, therefore it is very likely that other HDACi's such as TSA would similarly induce senescence in organoids.

However, the most important distinction is that VPA is widely used as a therapeutic drug, and has a strong record of causing (neuro)developmental birth defects, whereas TSA is not used in a similar manner. Therefore, while exposure to TSA might likely cause senescence and defects, the rationale to test this is not the same as for VPA.

Fig. 5 and 7 could be better integrated?

We appreciate this comment, and indeed in an earlier version of the manuscript (initial deposition on BioRxiv), we had the current Figs. 5, 6 and 7 integrated into one figure. However, we ultimately found this was too complex and led to a loss of some of the message. While we could integrate current Figs. 5 and 7 based on the fact that they both reference the phenotype of p19^{Arf}-deficient mice, we left the current Fig. 6 in the middle, as it documents the molecular profiling performed on E.D.9.5 mice, which is the same stages as shown in Fig. 5. Whereas then Fig. 7 is separate and shows the consequences at later stages, of the changes that were seen at E.D.9.5 as described in Figs. 5 and 6.

Reviewer #2: In this substantial report, Rhinn and colleagues use transgenic mice, electroporated chick embryos and human cerebral organoids to convincingly demonstrate that the anti-epileptic drug VPA causes p19ARf-mediated senescence of neuroepithelial cells. The comments below are intended to clarify aspects of the phenotyping and mechanistic links between induction of senescence and phenotypes observed.

We thank the reviewer for their insight, helpful suggestions, and appreciation of the novel findings and impact of the work. For the most part, we agree with the comments made, but in some places, we have slightly different opinions, as discussed.

1) The decreasing proportion of microcephalic embryos observed at E9.5-E13.5 in Fig 1B is perplexing but may be caused by misattribution at earlier stages. It is common for embryos with small open hindbrain or anterior neuropores to present with a smaller head due to lack of expansion of the presumptive ventricles. In many models, small cranial failures of neural tube closure expand as the tissue grow and become evident exencephaly at later stages. Misattribution is evidenced in the embryo labelled "Microcephaly" in Fig 2A, which has a midbrain opening visible in the images provided. The authors are encouraged to remove the phenotype data at E9.5/E10.5, leaving the more definitive E13.5 data. Quantitative analysis of head size relative to embryo body size is also essential to validate the microcephaly phenotype rather than a general stunting of growth.

We appreciate the insight and attention to detail from the reviewer, and acknowledge their comments on how small cranial failures of neural tube closure may, in some cases, lead to exencephaly at later stages. Of course, it is unfortunately not possible to track the same embryos over time to make conclusive claims. In our initial assessment, we classified embryos according

to gross appearance: a large failure in NT closure was called exencephaly, whereas a closed NT, but small forebrain/midbrain, was labeled microcephaly.

There were cases where embryos had both, an aberrantly open NT and a smaller brain; in such cases these were included in "exencephaly" counting. However, yes, there were also cases where the forebrain/midbrain was clearly smaller, but where the NT may have appeared as not being completely closed, as the highlighted case in Fig 2A. In cases such as this example, we did not count this opening in the NT as aberrant, as it likely would have closed if allowed to develop further, and we also examined to ensure the forebrain/midbrain was closed. But as the reviewer points out, yes, it is technically possibly that in some embryos, this may become exencephalic at later stages. Therefore, we had to set selective limits by which we classified the embryos, and we appreciate the reviewers point how this could lead to misattribution at these earlier stages. We believe that this would not lead to major changes in our quantification, but understand how it is somewhat selective and could imply a bias of changing dynamics across stages (e.g. that the incidence of microcephaly decreases).

To address this, we have followed the reviewer's suggestion, and we have removed the stacked bar graph depicting the quantification of the phenotypic differences at the earlier stages of E9.5 and E10.5. In addition, we have relabeled the exencephaly and microcephaly embryos as "open brain" and "small brain" respectively. And in the text, we have made efforts to rationalize the description, that we believe the more definitive phenotypes at E13.5 of exencephaly and microcephaly possibly arise from these early changes. Unfortunately, we feel that this removal of quantification now leaves a gap in the readers understanding of how penetrant these phenotypes are, so we include the "percent incidences" as approximate quantification at E9.5 in the text (not in the figure) to provide some perspective. In addition, we have replaced the highlighted embryo in Fig. 2A with a more representative one where the NT is fully closed, but still exhibits the same senescence pattern.

Finally, we maintain the label of exencephaly and microcephaly at E13.5, as these phenotypes are more definitive. We acknowledge the reviewers comment that ratio of head size relative to body size could support microcephaly nomenclature. However, we also point out that there are cases of microcephaly with dwarfism and smaller embryo size, or other major defects, that are still classified as microcephaly. And, as we discuss more below, the fact that we can rescue the E13.5 microcephaly phenotype independently of the somite and small body defects further supports the independence of these features, and that the term microcephaly is appropriate.

2) Please use the E9.5/E10.5 data to provide standard measures of embryo development such as somite number, dorsal length, turning score, etc. The authors may also wish to comment on secondary phenotypes such as hypoplastic pharyngeal arches visible in Fig 1B, which are also potentially relevant to fetal valproate syndrome. Note that cranial neural tube closure is not reliable completed before the ~17 somite stage in C57Bl/6J embryos so only embryos with >17 somites should be assessed for this phenotype (e.g. the WT control in Fig S4 with an open cranial NT has fewer than 17 somite, as does the p16KO labelled as Microcephaly in the same figure but which actually has an open cranial NT clearly visible at the apex of the head).

As the reviewer has suggested, we have now added standard measurements including somite number and dorsal length (Supp. Fig. 1), and we mention in the text that 100% of embryos, either control or VPA-treated had turned. In some examples, it may look like this is not completed, but this is an effect of the drastic somite defects in the posterior embryo. As can be seen, all WT embryos at the stages examined had more than 17 somites (both for the CD1 embryos in Fig. 1 and the C57/Bl6J embryos in Fig. 5) These graphs are now included as Supp. Figs. 1 and 7.

Regarding the WT control shown in Supp. Fig. 4, here we respectfully disagree with the reviewer's observation, and point out that the example shown in the figure actually had 18 somites (indicated here below). However, we agree this was not the best example, and some of the somites were not clear, or were obscured with membranes. Therefore, we have changed the example in the figure to avoid confusion.

Former Supp. Fig 4, WT Control embryo, with a circle to denote each somite.

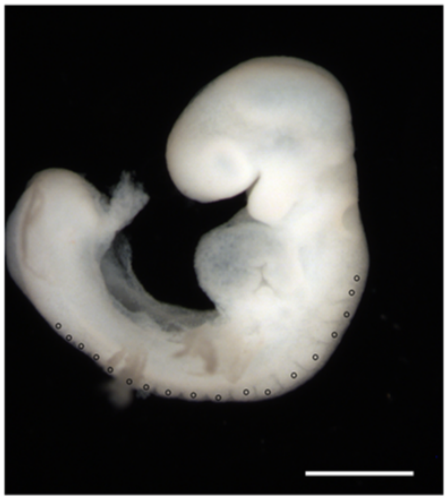

What becomes even more obvious with this new quantification is how treatment with VPA alters proper somite patterning and embryo length. It is clear that in the VPA treated embryos, there is massive impairment, fusion, or absence of somite development. Of course, now in the quantification of these embryos, it appears as if many of these have fewer than 17 somites. However, we highlight that what the graph actually represents is the number of intact / quantifiable somites per embryo. The embryos treated with VPA appear to have fewer somites because there were fewer intact and distinguishable somites to count. Interestingly, this phenotype appears to be more severe in the small brain embryos. As the litters were all chosen for treatment with VPA at random, then we can be certain that the litters were all initially at similar stages of development. We have more clearly annotated the images of the malformed somites, and include new images of the somite malformation in p19KO embryos (Fig. 1 and Supp. Fig 7), and commented on these in the text, as part of the secondary phenotypes. As is discussed below, the fact that these did not stain for SA-β-Gal, and that they were not rescued by p19-deficiency, we can conclude these are not senescence-related.

Finally again, and as discussed in response to point 1 above, we do acknowledge the reviewers concern that some of our examples shown may have what appears to be a small opening persisting in the apex. In our previous assessment, such small gaps were assumed to be within the normal range, and were not in the forebrain/midbrain area, and therefore were not considered as aberrant. When assessing exencephalic / open brain embryos, we only included those that were clearly beyond the normal range, and which likely would never have closed. Of course, we appreciate this is a difficult parameter to assess. We have now replaced the specific p16KO embryo highlighted with one that more accurately reflects the phenotype (Supp. Fig. 7).

3) The concentrations of valproic acid used in culture are rather high. No information is given on whether this substance was buffered prior to addition to culture (please also indicate the

catalogue number of the product purchased as Sigma offer various formulations). What was the pH of the culture medium after addition of 2 mM VPA?

We agree that the concentration of Valproic acid used is high. Our rationale was to use a higher dose, to try identify whether senescence was induced on a broad scale, and if so, where. Now that we have seen this, and in a surprisingly restricted pattern in the neuroepithelial cells, in future studies, we can try combinations of longer-term exposure and lower doses, to look for what might likely be a lower, but longer incidence of senescence induction. But this would potentially have been difficult to identify without this first approach.

We have now included in the methods, additional information regarding the VPA solution used in culture, including the catalog number of the product, and stating that this was not buffered prior to use. As suggested by the reviewer, we measured the pH of the culture medium after adding VPA. As can be seen below, addition of the VPA did not significantly change the pH of the medium, either before or after being placed in the CO_2 incubator.

PH Measurement:

Medium at Room Temp

Ctrl :	*pH=7,43*
1mM VPA :	*pH=7,44*
2mM VPA :	*pH=7,42*

Medium at 37°C in the incubator

Ctrl :	*pH=7,35*
1mM VPA :	*pH=7,34*
2mM VPA :	*pH=7,37*

4) It is convincing that the VPA-exposed organoids are smaller. It is therefore not surprising that the neural rosette surface and Tuj1 thickness are smaller in the treated organoids. Please normalise these to a measure of organoid size to clarify whether VPA largely acted to restrict neuroepithelial cell expansion (as suggested by other data in the manuscript) or also subsequently impaired neural differentiation.

As we showed in the original submission, organoids are smaller at days 25 and 42 after VPA exposure, and both neuroepithelial (Pax6) and neuron (Tuj1) area is significantly reduced in response to both 1 and 2mM VPA, when measured at day 42. It is true that as the overall organoids are smaller in response to the VPA, then maybe it is possible that the rosettes are smaller as a result of an overall decrease in size. Here however, we would argue that organoid growth parameters are not fully clear, and as far as we are aware, it has not been determined whether the rosettes and internal connective tissue can grow independently of each other – indeed, it is more likely that proper rosette expansion is needed and contributes as a primary size determinant to the total organoid size, rather than vice versa. Therefore, any perturbation of rosette development, would likely lead to a decreased overall organoid size. Therefore, we feel that the best comparison is, as we originally showed, to compare rosette size and Tuj1 thickness between conditions. Similar measurements were used in a recent landmark study that used a screening approach in organoids to study microcephaly (e.g. Esk et al, Science, 2020; https://www.science.org/doi/10.1126/science.abb5390).

However, as the reviewer suggested, we did the requested comparisons, normalizing neural rosette surface area to overall organoid size, and then Tuj1 thickness to rosette thickness (shown below). Here, it can be seen that the ratio of rosette size to total organoid size is similar for 1mM VPA, but decreases slightly with 2mM VPA, suggesting there is only a small effect on neuroepithelial cell expansion at high dose. However, when we compared Tuj1 thickness to rosette thickness (we cannot reliably compare Tuj1 <u>thickness</u> to organoid <u>area</u>), this appears to show that there is a stronger effect on neuronal differentiation. Again, however, we do not feel this is the best way to represent this data. In addition, as in mice, we clearly show a decrease in NE cell proliferation as well as differentiation, so we think both processes are affected and directly linked.

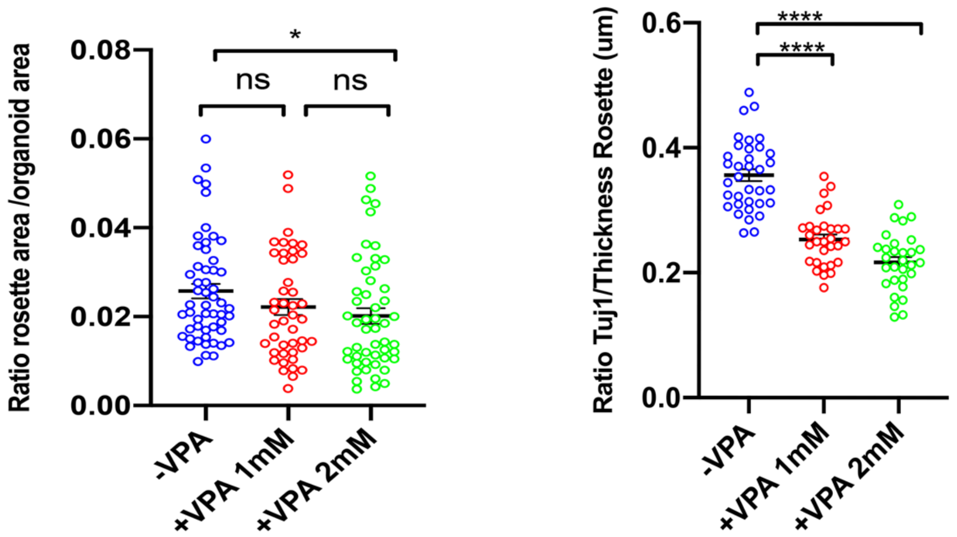

5) Fig 5A: The apparent exacerbation of exe induced by VPA in p21 and p16 KO is striking. Non-VPA treated controls need to be provided to support interpretation of this data. This data is limited because it was performed at E9.5 when the distinction between exe and microcephaly is in doubt (comment 1). Nonetheless it may suggest that p21/p16 expression is protective, enabling the embryo to convert the fatal exe phenotype into viable microcephaly. In this E9.5 cohort, were other features of embryo development such as somite gain also rescued by p19 KO or was the effect limited to the cranial neural tube?

We appreciate the reviewer noticing that there is a possible exacerbation of the exencephaly phenotype in the p21 and p16 KO following VPA exposure. We are also intrigued, as this implies that these genes may act in a protective manner in response to such damage. However, as the reviewer also points out, this is based on the E9.5 stages, and we have not yet been able to assess whether this manifests similarly at later stages (it is difficult to get large numbers of p16 KO embryos). In light of the reviewers combined comments (related to point 1 above and this current point), as we have removed the phenotypic quantification from Figure 1, we also therefore have removed the phenotypic quantification of p19, p16, and p21 KO embryos from former Fig. 5A, as these analyses were based on E9.5 data. Unfortunately, one consequence of this is that we lose the potentially interesting result that p21 and p16 deficiency exacerbates the exencephaly phenotype. However, as this is not the main point of this study, and would need to be extensively validated at later stages, we feel this is more suitable for a future study.

However, to quantitatively support our initial observations, we have now included the measurements of forebrain/midbrain size for p21 and p16 KO embryos, as we had originally included for p19 KO (New Fig 5A). This further supports our original claims, that there was noticeable effect of p19-deficiency on VPA-induced small brain phenotype, that was not present in p21 and p16-deficient embryos. As the reviewer also correctly pointed out, we had omitted the non-VPA treated controls for each genotype. We have now included these for p21 and p16 KO, showing that these are similar to WT, in terms of the assays used, and do not exhibit any of the phenotypes in the absence of treatment (Fig. 5A and Supp. Fig. 6).

Regarding the rescue of phenotype by p19-deficiency, we have had previously observed and mentioned that only the head phenotype was rescued. However, we have now quantified this to show that in p19 KO, while there is a significant rescue in the size of the forebrain/midbrain (and associated senescence), there is no rescue in the trunk phenotypes of abnormal somite development, or overall length of the embryo. These measurements have now been included in Supp. Fig 7. This indeed shows that the effects on the forebrain/midbrain are independent of the effects on the trunk. This figure also includes examples of untreated p19KO as controls, showing they are phenotypically normal in the absence of treatment.

6) The apical localisation of B-gal stained cells in the neuroepithelium in vivo is striking. Can the authors comment on the distribution of these cells? Restricted apical distribution could be an artefact if the embryos were stained in wholemount before sectioning (as described in the methods) due to limited penetration of B-gal. This is an important consideration given the exposed NE or smaller heads of the VPA-treated embryos will enable greater reagent access (this reviewer is convinced that senescence really is increased, but potentially not to the extent and localisation suggested by the images).

This is an important question, as the apical localization of the *SA-β-Gal* staining is indeed quite striking. In the figures shown, yes, the embryos were stained in wholemount and were subsequently sectioned. In our experience with staining embryos for *SA-β-Gal activity*, this pattern of staining would not likely be an artefact, as the penetration of the staining reagent at these early stages is quite high. However, in response to the reviewer's question, we repeated the experiments of exposing the embryos to VPA, and then cut frozen sections which were then subsequently stained with *SA-β-Gal*. As can be seen in the example shown below, the apical pattern of staining was identical to that seen by wholemount. However, the histology is not as good, so we therefore chose to leave the original images in the main figure. We now include a statement in the text that a similar pattern of staining was seen with wholemount or staining on sections.

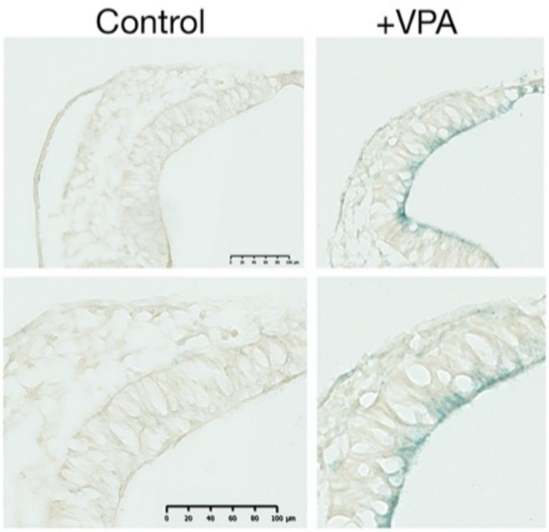

Figure: SA-β-Gal staining on sections: Embryos were embedded in OCT, and cryo-sections were subsequently stained for SA-β-Gal activity. This approach shows a similar apical localization as when embryos were subjected to wholemount staining.

7) SOX1/apical area is quantified in the organoid system, whereas Pax6 and Tbr2 are quantified in mice. Can the same parameters be provided in both systems?

Essentially, the same parameters are provided for both systems, but different antibodies were used, based on availability and compatibility.

To explain, anti-Tbr2 (rat) and anti-Pax 6 (rabbit) were used in the mouse samples, to label the intermediate progenitors and apical progenitors respectively. Unfortunately, the anti-Tbr2 (rat) does not work on human samples, so we could not use this on organoids. We have another anti-Tbr2 (rabbit) which we used in humans, but which could not be used for co-staining with Pax6, because both are rabbit-produced antibodies. Therefore, we used Sox1, which is another marker of apical-progenitors, similar to Pax 6.

To support this switch, we performed additional staining (shown here below, in Day 25 and Day 42 organoids), co-staining for Pax6 and Sox1 in human organoids to show that they label the same population of cells. Therefore, we feel that both human and mouse analyses are comparable.

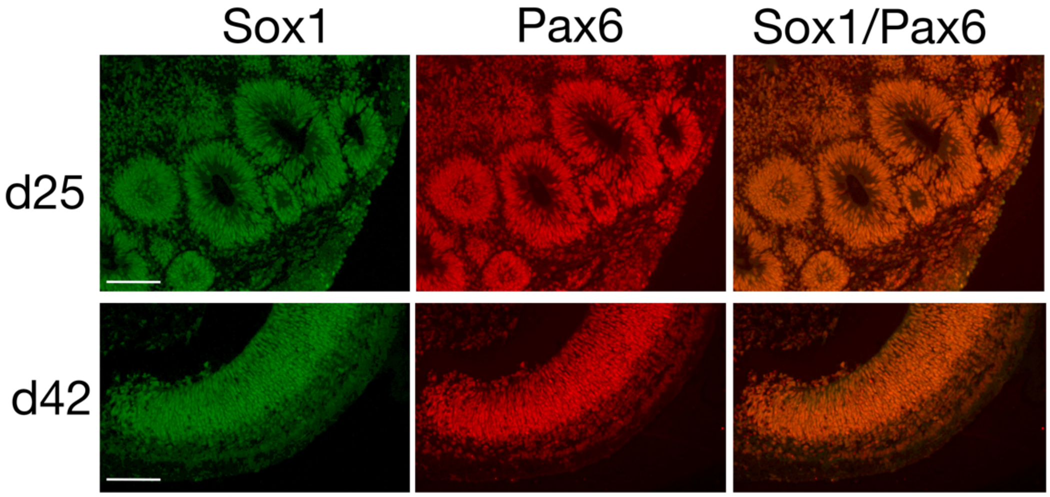

8) Does the RNASeq dataset provide any insights into whether it is the presence of senescent cells, or the non-senescent cells' responses to SASP which underlies the phenotypes observed?

This remains a major question for future studies. As the reviewer alludes to, senescent cells can exert cell-intrinsic effects, blocking proliferation/differentiation of affected cells, or can induce changes in neighboring cells, via the SASP. Unfortunately, the bulk-RNA sequencing approach we used makes it impossible to distinguish between cell-intrinsic and cell-extrinsic effects. In future studies, we hope to perform single-cell sequencing analyses, which would be ideal to determine the cell populations that exhibit senescence gene-induction, and those which may be affected by this.

9) Fig 7: Were only microcephalic embryos included in the WT+VPA group, or did this include exe embryos with degenerating NE?

Yes, only microcephalic embryos were used in this figure. We have made this clearer in the text and figure legends.

10) The authors interpret the EdU assay as showing NE cells are "proliferative in control but not in VPA-exposed embryonic mice." This interpretation is not clear given the extensive number of EdU-positive, basally-located NE cells in both groups (Fig 2 C). EdU should label cells with basally-located nuclei as this is where S phase occurs. In the images provided it appears that in the control embryo some cells had progressed through the cell cycle whereas in the VPA-treated embryos most are still basal. Please quantify the EdU staining to support interpretation.

The reviewer is correct, and here we acknowledge we did not explain the results clearly. As the reviewer correctly points out, EdU staining will mostly label basal cells. However, it does still label *<u>some</u> cells in the apical area (e.g. closer to the dashed line in Fig. 2C). What we intended to say, and which we have now corrected in the text, is that the apical pattern of EdU was decreased in the VPA-treated embryos, generating a large zone without positive staining, which was identified with the red asterisk. Interestingly, this is the zone where SA-β-Gal localizes, as discussed above. We have been able to image some co-stained cells (SA-β-Gal and EdU) which supports that indeed, the senescent cells do not proliferate. However, the imaging of these doubly-labeled cells is sub-optimal, so we did not include it.*

In total, based on the reviewer's comments, we have modified the text to make it clear that the EdU staining shows that the apical cells have decreased proliferation, in the same approximate area where we see SA-β-Gal. In addition, as requested, we have quantified the EdU, representing this for total area, as well as apical staining. In both cases, there is a significant decrease in proliferation, which is now shown in Supp. Fig. 2A,B.

Spurred by this, we also performed additional experiments, this time staining with an anti-phospho-histone H3 (PHH3) antibody, which should stain proliferating apical cells. Indeed, what this clearly shows is that there is a significant decrease in apical proliferation in response to VPA (shown in Supp. Fig. 2C). Altogether, these data support and improve on our original results, that senescent cells do no proliferate, are apically located, and perturb normal proliferation patterns.

11) Similarly, the TUNEL staining is unconvincing without quantification. Although the authors interpret the images as showing increased positivity in the non-neural ectoderm, staining is also evident in the neural fold tips.

We agree that quantification of the TUNEL staining should be performed, especially if we wanted to make the claim that it is linked to exencephaly / open brain phenotypes. However, this was not the intention, so we have removed this statement. Our main point is to say that while there was visibly increased incidence in some regions of the embryo following VPA treatment, <u>there was no major induction of apoptosis in the NE cells where we see SA-β-Gal.</u> We now state this point more clearly in the text.

In addition, the reviewer is correct that TUNEL staining is evident in the neural fold tips, this is present both in treated and untreated embryos. However, we had unintentionally cropped this pattern of staining from the control embryos. We have retaken this image to show that TUNEL staining is detectable in the fold tips in each condition, and we mention this in the text. However, again, this is independent of our primary claim, that there is no increase in cell death in the NE cells, where SA-β-Gal is detected.

12) Were all organoid studies performed with a single cell line?

Yes, all the organoid studies were performed in a single cell line. Repeating the findings in an additional cell line would have been prohibitively expensive and time-consuming. We had chosen a cell line that has been validated in the organoid field, representing the early stages of neurodevelopment as best as is currently possible. This cell line, HPSI0214i-kucg_2, has been extensively characterized, both by the HipSci Consortium from whom we purchased the line, (https://www.hipsci.org/lines/#/lines/HPSI0214i-kucg_2), and in the literature (e.g. Kanton et al, Nature 2019, https://pubmed.ncbi.nlm.nih.gov/31619793/).

Reviewer #3: The manuscript by Rhinn and colleagues illustrates a novel role for cellular senescence in the context of embryonic development. The paper describes a new role for p19/p14 during development, which is detrimental for brain development.

This is a high quality paper, very well written and experiments are well conducted. Conclusions are fully supported by the data. I believe this paper should be published-I have no major suggestions to improve it.

1 minor comment: Figure 6 in C it says genes highlighted in E, but I guess the authors mean B

We thank the reviewer for their appreciation of the manuscript, and their time invested. We have taken note of the mis-labeling in the figure legend and have corrected this.
